# Repeated microdoses of LSD do not alter anxiety or boldness in zebrafish

**DOI:** 10.1038/s41598-024-54676-8

**Published:** 2024-02-22

**Authors:** Ethan V. Hagen, Melike Schalomon, Yanbo Zhang, Trevor J. Hamilton

**Affiliations:** 1 Department of Psychiatry, University of Alberta, Edmonton, AB Canada; 2 Department of Psychology, MacEwan University, 10700 104 Ave NW, Edmonton, AB T5J 4S2 Canada; 3 Neuroscience and Mental Health Institute, University of Alberta, Edmonton, AB Canada

**Keywords:** Neuroscience, Psychology, Pharmacology, Animal behaviour

## Abstract

The therapeutic use of lysergic acid diethylamide (LSD) has resurfaced in the last decade, prompting further scientific investigation into its effectiveness in many animal models. Zebrafish (*Danio rerio*) are a popular model organism in medical sciences and are used to examine the repeated administration of pharmacological compounds. Previous zebrafish research found acute LSD altered behaviour and cortisol levels at high (250 µg/L) but not low (5–100 µg/L) levels. In this study, we used a motion tracking system to record and analyze the movement patterns of zebrafish after acute and repeated 10-day LSD exposure (1.5 µg/L, 15 µg/L, 150 µg/L) and after seven days of withdrawal. The open-field and novel object approach tests were used to examine anxiety-like behaviour, boldness, and locomotion. In the acute experiments we observed a significant decrease in high mobility with 1.5 µg/L, 15 µg/L, and 150 µg/L of LSD compared to the control and a decrease in velocity with 1.5 and 15 µg/L. In repeated experiments, there were no significant differences in the levels of anxiety, boldness, or locomotion between all LSD groups and controls immediately after 10-day treatment or after withdrawal.

## Introduction

Lysergic acid diethylamide (LSD) is a psychedelic drug that can alter how the brain integrates and segregates information and can induce visual hallucinations and illusions, intensified thoughts, and emotions^1^. LSD belongs to a category of drugs known as classical hallucinogens, which interact with 5-HT receptors, primarily the 5 HT_1A_ and 5-HT_2A_ subtypes^2,3^ and can increase serotonin levels in the brain^4^. The hallucinogenic effects of LSD are presumed to be due to the activation of 5-HT_2A_ and also 5-HT_2C_ receptors^5^. The interaction of LSD on 5-HT_2A_ receptors leads to hyperactivity in the cortico-striatal–thalamo-cortical circuit which leads to thalamic gating sensory information resulting in hallucinations^6^. However, the effects of LSD on the brain and psychological processes are not limited to its hallucinogenic effects^1^. ‘Microdosing’ involves repeatedly taking LSD or other psychedelic drugs in small quantities to obtain sub-hallucinogenic positive effects on wellbeing. A microdose is typically around ten percent of the regular psychedelic dose^7^. Microdosing psychedelics, like psilocybin, may have potential in the treatment of psychiatric disorders, but further research is needed to better understand the impact of microdosing on treatment outcomes^8^. The majority of human-based microdosing studies used self-report methods. Anecdotally, microdosing was associated with an improved quality of life^9^. An online survey found that microdosing psychedelics reduced negative emotionality and dysfunctional attitude, and increased wisdom, open-mindedness, and creativity^10^. In contrast, a randomized control trial reported that a single dose of LSD at 26 μg increased vigor and reduced the positive rating of images tied to positive emotions, compared to lower microdoses at 6.5 and 13 μg, suggesting LSD has a dose-dependent impact on psychological experiences in healthy participants^11^. However, the side effects of microdosing psychedelics remain unclear. One animal study showed that microdosing psilocybin, not LSD, prevented alcohol use relapse but only for a short duration with no long-term benefits^12^. Given the potential of psychedelics in managing a variety of psychiatric disorders, further studies are required to outline the behavioural patterns in different model organisms to understand the impact on neurobiological processes.

Pioneering research on the effects of LSD in fish occurred using Siamese fighting fish (*Betta splendens*)^13^ and behavioural impacts were compared to other fish, like the goldfish^13^. A dose of 0.5 µg/ml resulted in an increase in surface preference behaviour in both species. Another early study tested several tropical fish: Siamese Fighting Fish, White Clouds (*Tanichthys albonubes*), and Guppies (*Lebistes reticulatus*). Fish were pre-treated using DL-3-[3,4-Dihydroxyphenyl] alanine (DOPA), tryptamine, and tryptophan, then tested for overall activity and food-seeking behaviour after one hour of LSD exposure, with different dosages for the different fishes; 1 µg/ml for the White Cloud and the Siamese Fighting Fish, and 4 µg/ml for the Guppies^14^. All species of fish showed a darkening of skin colour, increase in time at surface, less anxiety-like behaviour towards a probe, and a general decrease in activity with less interest in food. In another study, large carp (species not specified) exhibited more restlessness and increase in surface preference behaviour compared to control fish after an LSD treatment^15^. Observations from these historical studies suggest there is a dose-dependent anxiolytic (anxiety-reducing) effect of LSD requiring further study.

Zebrafish (*Danio rerio*) are a recently emerging and reliable model organism for many studies, including pharmacological experiments^16^. They can reliably be tested with anxiety-like behavioural paradigms such as light–dark preference, shuttle box avoidance, shoaling, and alarm reaction tests^17^. Zebrafish can also readily absorb water-soluble chemicals, thus allowing for ease of administration when conducting pharmacological research^18^. To date there have been few studies with LSD and zebrafish. The most thorough zebrafish study on LSD observed behavioural changes with the highest dose, 250 µg/L, but not with 5–100 µg/L. This study used a multitude of behaviour paradigms, including the open field (OF) test, light–dark preference text, shoaling and social behaviour tests, T-maze tests, novel tank tests, and an observation cylinder cycle to examine the behavioural effects of LSD on the fish^19^. The main findings were less immobility and more time transitioning between zones with the highest dose (250 µg/L). Another study examining the impact of LSD on zebrafish shoaling found a 100 µg/L dose significantly increased shoal size, consistent with an anxiety-reducing effect^20^. Therefore, LSD can impact anxiety-like behaviour and activity levels in the zebrafish with an acute exposure.

There are evident effects of acute doses of LSD on anxiety-like behaviour^19,20^, but microdosing over multiple days has not been studied in zebrafish. This study investigated the effects of repeated microdosing sessions in zebrafish at concentrations of 1.5 µg/L, 15 µg/L, 150 µg/L, given for 30 min per day for 10 consecutive days. In another experiment we assessed the impact of these doses given acutely for 30 min. Anxiety-like behaviour, boldness, and locomotion were tested using an OF and novel object approach (NOA) test immediately after acute exposure, repeated dosing, and after withdrawal from repeated dosing.

## Results

Behavioural responses to acute LSD exposure at 0, 1.5, 15, and 150 µg/L were examined with the OF and NOA tests. For repeated dosing experiments, fish were dosed for 10 days at either 0, 1.5, 15, and 150 µg/L of LSD, then tested on the last day of dosing and again 7-days later. For both OF and NOA, the velocity, time in the transition zone, time in the inner zone, time in thigmotaxis, meandering, high mobility, low mobility, and overall mobility were quantified. Sex differences were analyzed for all variables and only one (1.5 µg/L dose in the high mobility variable in the repeated exposure post dose group) showed a sex difference, however, this was not considered meaningful due to the high number of comparisons that were not significant (Data in supplemental materials).

### Open field—acute exposure

**Figure 1:**
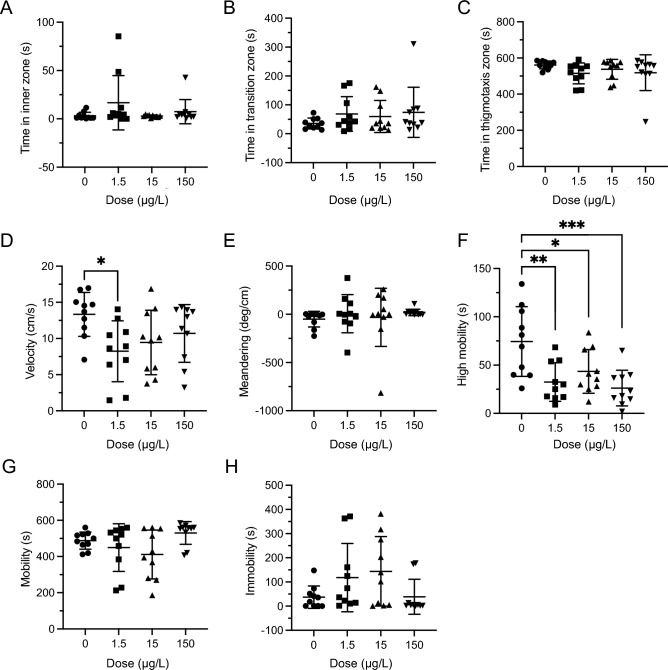
Acute LSD effect in the open field (OF) test. (**A**) Time spent in inner zone was not significantly different after acute dosing. (**B**) Time in transition zone was not significantly different after acute dosing. (**C**). Time spent in thigmotaxis zone was not significantly different after acute dosing. (**D**) Velocity was significantly different after acute dosing. The * represents a significant difference of *P* < 0.05. (**E**) Meandering was not significantly different after acute dosing. (**F**) High mobility was significantly different after acute dosing. The * represents a significant difference of *P* < 0.05, ** represents a significant difference of *P* < 0.01, and *** represents a significant difference of *P* < 0.001. (**G**) Mobility was not significantly different after acute dosing. (**H**) Immobility was not significantly different after acute dosing. Sample sizes for A-H: 0 (n = 10), 1.5 (n = 10), 15 (n = 10), 150 (n = 10). Data presented as mean + /- SD.

There were no significant effects of LSD on the time fish spent in the inner, transition and thigmotaxis zones (*Inner*:* H*(3) = 3.901,* P* = 0.2724 Fig. 1A; Transition: *H*(3) = 1.939,* P* = 0.5852, Fig. 1B; Thigmotaxis: *H*(3) = 5.243,* P* = 0.1549, Fig. 1C). There was a significant difference in velocity (*F*(3,36) = 3.036, *P* = 0.0415, Fig. 1D) with post-hoc differences between control and 1.5 µg/L group (*P* = 0.0323). Meandering was not significantly different between any of the doses (*H*(3) = 4.976, *P* = 0.1736, Fig. 1E). High mobility was significantly different (*F*3,36 = 7.184, *P* = 0.0007, Fig. 1F) with all dosed groups showing a decrease in high mobility compared to the control group (0 µg/L vs 1.5 µg/L *P* = 0.0037, 0 µg/L vs 15 µg/L *P* = 0.0450, 0 µg/L vs 150 µg/L *P* = 0.008). There was no significant difference present in the mobility parameter (H(3) = 7.699, *P* = 0.0527, Fig. 1G). Immobility did not yield any significant differences between any of the doses (*H*(3) = 6.169, *P* = 0.1037, Fig. 1H).

### Novel object approach—acute exposure

**Figure 2:**
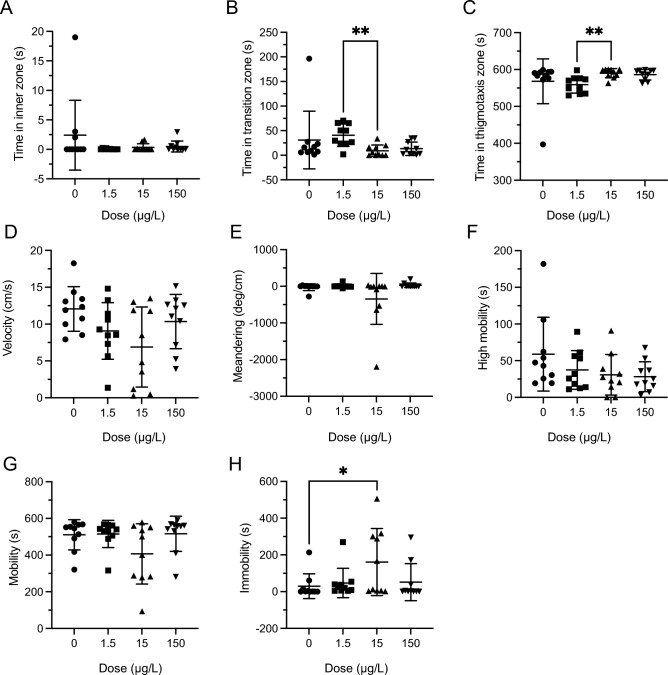
Acute LSD effect in the novel object approach (NOA) test. (**A**) Time spent in inner zone was not significantly different during acute dose NOA testing. (**B**) Time in transition zone was significantly different between groups during acute dose NOA testing. The ** represents a significant difference of *P* < 0.01. (**C**). Time spent in thigmotaxis was significantly different between groups during acute dose NOA testing. The ** represents a significant difference of *P* < 0.01. (**D**) Velocity was not significantly different during acute dose NOA testing. (**E**) Meandering was significantly different during acute dose NOA testing, but there were no post-hoc differences. (**F**) High mobility was not significantly different during NOA acute dose testing. (**G**) Mobility was not significantly different during the acute dose NOA testing. (**H**) Immobility was not significantly different during NOA acute dose testing with a difference between 0 µg/L and 15 µg/L. The * represent a significant difference of *P* < 0.05. Sample sizes for A-H: 0 (n = 10), 1.5 (n = 10), 15 (n = 10), 150 (n = 10). Data presented as mean + /- SD.

There were no significant effects of LSD on the time fish spent in the inner zone (Fig. 2A; *H*(3) = 0.750, *P* = 0.8614). However, LSD significantly altered the time fish spent in the transition zone (Fig. 2B; *H*(3) = 12.01, *P* = 0.0074), with post-hoc differences between the 1.5 and 15 µg/L groups (*P* = 0.0046). LSD also significantly changed the time fish spent in the thigmotaxis zone (Fig. 2C; *H*(3) = 12.05, *P* = 0.0072) with post-hoc differences between the 1.5 and 15 µg/L groups (*P* = 0.0046). Velocity was not altered by any LSD dose (Fig. 2D; *F*(3,36) = 2.816, *P* = 0.0529). There was a significant main effect of LSD on meandering (*H*(3) = 7.819, *P* = 0.0499, Fig. 2E), but no post-hoc significant differences. LSD had a no significant effect on high mobility (*H*(3) = 3.378, *P* = 0.2912, Fig. 2F). Mobility was not altered by LSD treatment (*H*(3) = 4.251, *P* = 0.2356, Fig. 2G). LSD did significantly alter immobility (*H*(3) = 9.436, *P* = 0.0240, Fig. 2H) with post-hoc differences between 0 µg/L and 15 µg/L groups (*P* = 0.0498).

### Open field test—repeated exposure—post dose

**Figure 3:**
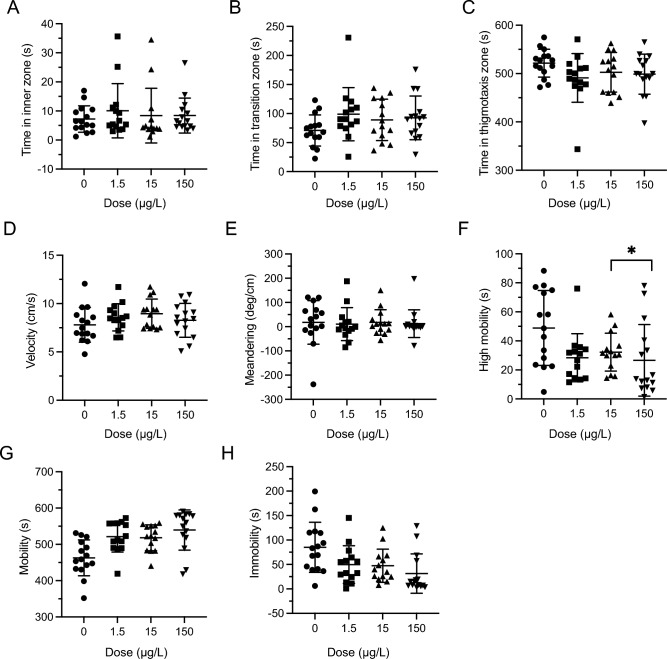
Repeated LSD effect in the open field (OF) test after last repeated dose. (**A**) Time spent in the inner zone was not significantly different during post-dose OF testing. (**B**) Time in the transition zone was not significantly different during post-dose OF testing. (**C**). Time spent in the thigmotaxis zone was not significantly different during post-dose OF testing. (**D**) Velocity was not significantly different during the post dose OF testing. (**E**) Meandering was not significantly different during post-dose OF testing. (**F**) High mobility was significantly different between dosing groups but not from control during post-dose OF testing. The * represents a significant difference of *P* < 0.05. (**G**). Mobility was not significantly different during post-dose OF testing. (**H**) Low mobility was not significantly different during post-dose OF testing. Sample sizes for A-H: 0 (n = 54), 1.5 (n = 53), 15 (n = 54), 150 (n = 54). Data presented as mean + /- SD.

There were no significant differences for the time spent in zones between any LSD groups and controls (Inner: *F*(3,12) = 0.2819, *P* = 0.8375, Fig. 3A; Transition: *F*(3,211) = 0.9908, *P* = 0.3981, Fig. 3B; Thigmotaxis: *F*(3,12) = 0.7793, *P* = 0.5279 Fig. 3C). Within the set of locomotor variables there were also no significant differences across any groups (Velocity: *F*(3,211) = 0.5231, *P* = 0.6669, Fig. 3D; Meandering: *F*(3,12) = 0.4375, *P* = 0.7302; Fig. 3E). There was a significant difference in high mobility (*F*(3,12) = 2.900, *P* = 0.0360; Fig. 3F) with post-hoc differences between 15 µg/L and 150 µg/L (*P* = 0.0230) but no difference from control. There were no significant differences in mobility (Mobility; *F*(3,12) = 2.688, *P* = 0.0935: Fig. 3G) or immobility (Immobility: *F*(3,12) = 2.392, *P* = 0.1261, Fig. 3H).

### Novel object approach test—repeated exposure—post dose

**Figure 4:**
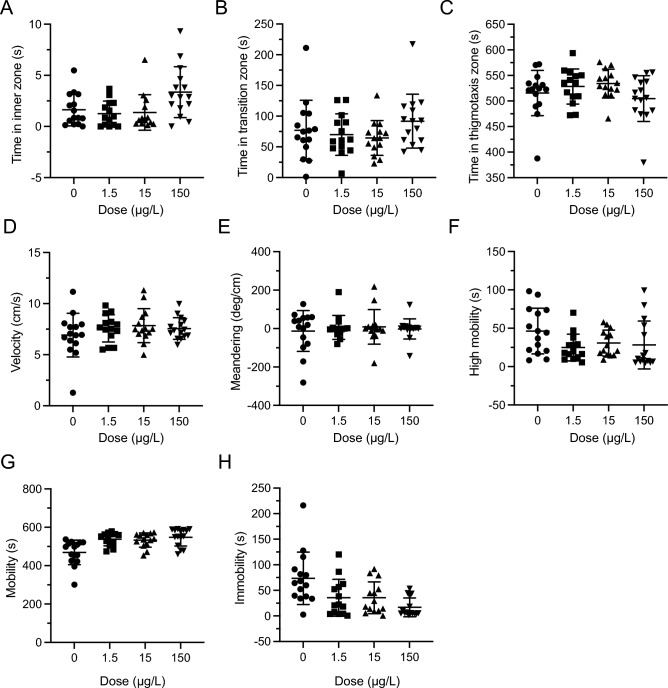
Repeated LSD effect in the novel object approach (NOA) test after last repeated dose. (**A**) Time spent in the inner zone was not significantly different during post-dose NOA testing. (**B**) Time spent in the transition zone was not significantly different during post-dose NOA testing. (**C**). Time spent in the thigmotaxis zone was not significantly different during post-dose NOA testing. (**D**) Velocity was not significantly different during the post dose NOA testing. (**E**) Meandering was not significantly different during post-dose NOA testing. (**F**) High velocity was not significantly different during post-dose NOA testing. (**G**). Mobility was not significantly different during post-dose NOA testing. (**H**) Low mobility was not significantly different during post-dose NOA testing. Sample sizes for A-H: 0 (n = 54), 1.5 (n = 53), 15 (n = 54), 150 (n = 54). Data presented as mean + /- SD.

The time spent in zones were not significantly changed by LSD (Inner: *F*(3,210) = 1.210, *P* = 0.3069, Fig. 4A; Transition: *F*(3,210) = 0.9408, *P* = 0.4218, Fig. 4B; Thigmotaxis: *F*(3,210) = 0.8788, *P* = 0.4529, Fig. 4C). Similarly, velocity, meandering, high mobility, mobility and immobility were not significantly different between any of the experimental groups (Velocity: *F*(3,12) = 0.9112 *P* = 0.4645, Fig. 4D; Meandering: *F*(3,12) = 0.5810, *P* = 0.6387, Fig. 4E; High mobility: *F*(3,12) = 0.9773, *P* = 0.4357, Fig. 4F; Mobility: *F*(3,12) = 1.967, *P* = 0.1728, Fig. 4G; Immobility: *F*(3,210) = 0.2376, *P* = 0.8701, Fig. 4H).

### Open field—repeated exposure—withdrawal

**Figure 5:**
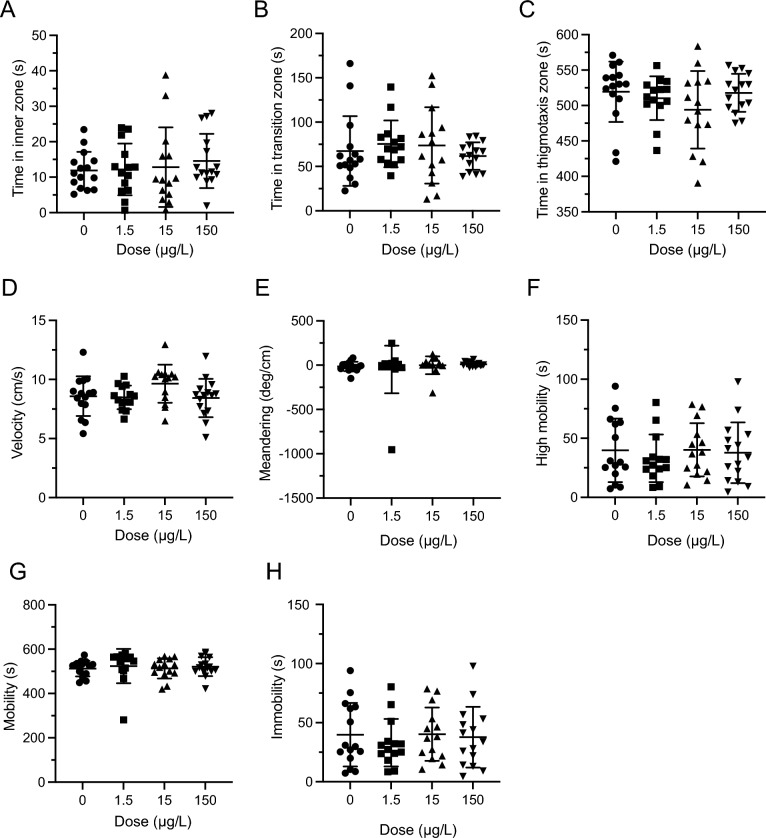
Repeated LSD effect in the open field (OF) test during withdrawal. (**A**) Time spent in the inner zone was not significantly different during withdrawal OF testing. (**B**) Time spent in the transition zone was not significantly different during withdrawal OF testing. (**C**). Time spent in the thigmotaxis zone was not significantly different during withdrawal OF testing. (**D**) Velocity was not significantly different during the withdrawal OF testing. (**E**) Meandering was not significantly different during post-dose OF testing. (**F**) High mobility was not significantly different during withdrawal OF testing. (**G**). Mobility was not significantly different during withdrawal OF testing. (**H**) Low mobility was not significantly different during withdrawal OF testing. Sample sizes for A-H: 0 (n = 54), 1.5 (n = 52), 15 (n = 54), 150 (n = 54). Data presented as mean + /- SD.

There were no significant differences in time spent in all three zones between any of the groups (Inner: *F*(3,12) = 0.9821, *P* = 0.4337, Fig. 5A; Transition: *F*(3,12) = 0.5804, *P* = 0.6390, Fig. 5B; Thigmotaxis: *F*(3,210) = 0.2167, *P* = 0.8847, Fig. 5C). These results suggest that LSD does not affect the zone preference of zebrafish after the withdrawal period. Meandering and the mean velocity (cm/s) showed no significance across any of the groups (Velocity: *F*(3,12) = 1.558, *P* = 0.2507 Fig. 5D; Meandering: *F*(3,12) = 0.7181, *P* = 0.5600, Fig. 5E). None of the mobility variables showed a significant difference between the experimental groups (High mobility: *F*(3,210) = 0.3176, *P* = 0.8126, Fig. 5F; mobility: *F*(3,12) = 0.07132, *P* = 0.9742, Fig. 5G; Immobility: (*F*(3,210)) = 0.3176, *P* = 0.8126 Fig. 5H). Overall, all variables tested showed that none of the LSD doses had a significant effect after the 7-day withdrawal period.

### Novel object approach—repeated exposure—withdrawal

**Figure 6:**
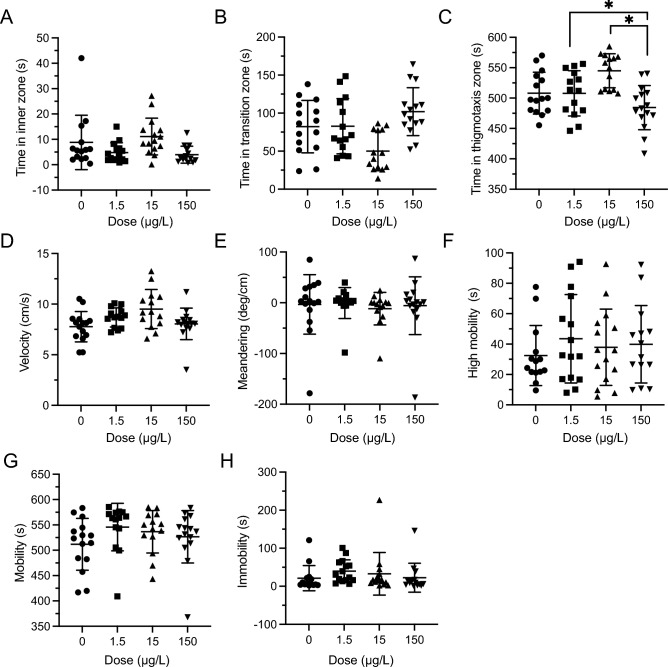
Repeated LSD effect in the novel object approach (NOA) test during withdrawal. (**A**) Time spent in inner zone was not significantly different during withdrawal NOA testing. (**B**) Time in transition zone was not significantly different during withdrawal NOA testing. (**C**). Time spent in thigmotaxis was significantly different between LSD groups during withdrawal NOA testing. The * represents a significant difference of *P* < 0.05. (**D**) Velocity was not significantly different during the withdrawal NOA testing. (**E**) Meandering was not significantly different during withdrawal NOA testing. (**F**) High velocity was not significantly different during withdrawal NOA testing. (**G**). Mobility was not significantly different during withdrawal NOA testing. (**H**) Low mobility was not significantly different during withdrawal NOA testing. Sample sizes for A-H: 0 (n = 54), 1.5 (n = 52), 15 (n = 54), 150 (n = 54). Data presented as mean + /- SD.

There was no significant difference in the time in the inner zone for any four groups (Inner: *F*(3,210) = 1.580, *P* = 0.1953, Fig. 6A). However, the time spent in thigmotaxis, and time spent in the transition zone was significantly different (Transition: *F*(3,210) = 2.832, *P* = 0.0393, Fig. 6B; thigmotaxis: *F*(3,210) = 3.950, *P* = 0.0091, Fig. 6C). Despite the main effect, a Tukey’s multiple comparison found no difference in the transition zone when the groups were compared. But in thigmotaxis zone, 1.5 µg/L vs. 150 µg/L (*P* = 0.0162) and 15 µg/L vs. 150 µg/L (*P* = 0.0221) were significantly different with post-hoc testing. Meandering, velocity and all forms of mobility showed no significant differences when tested after the withdrawal period (Velocity: *F*(3,12) = 0.6587, *P* = 0.5930, Fig. 6D; Meandering: *F*(3,12) = 0.08749, *P* = 0.9656 Fig. 6E; High mobility: *F*(3,210) = 1.461, *P* = 0.2263, Fig. 6F; mobility: *F*(3,12) = 1.315, *P* = 0.3150 Fig. 6G; Immobility: *F*(3,210) = 0.6423, *P* = 0.5886 Fig. 6H).

## Discussion

The aim of this study was to investigate the effects of a 10-day LSD microdosing schedule on anxiety-like behaviour, boldness, and locomotion in zebrafish. Behavioural data was collected following the last dose as well as after a 7-day withdrawal period. There were no significant differences in locomotion, anxiety-like behaviour or boldness in the LSD groups compared to the controls after the last dose. Significant differences were found after the withdrawal period in the NOA for one parameter, but they were between LSD groups and not with the controls. To validate previous research demonstrating an acute effect of LSD, we also performed and acute experiment and found a dose-dependent sedative effect. Taken together, the effect of repeated LSD microdosing on anxiety-like behaviour, boldness, and locomotion is negligible under our experimental conditions.

Acute effects of LSD in fish have been observed in previous studies in various species of fishes. The Siamese Fighting Fish (*Betta splendens*), White Clouds (*Tanichthys albonubes*), and Guppies (*Lebistes reticulatus*)^14^ responded with behavioural changes from acute LSD exposure. There were a multitude of effects following LSD exposure, but relevant here is a decrease in overall activity^14^, consistent with the decreased high mobility we found in zebrafish. In our acute dose experiment, LSD had a sedative effect at all 3 doses (1.5 µg/L, 15 µg/L, 150 µg/L) compared to control fish. This is inferred from a decrease in ‘high mobility’ across all doses. We also observed a decrease in velocity with 1.5 and 15 µg/L consistent with a sedative effect. In other zebrafish studies, anxiolytic effects of LSD were observed in group shoaling^20^ and in the OF, novel tank dive, T-maze, and social preference test^19^. These acute effects contrast with the lack of effects we observed on anxiety-like behaviour and boldness but are likely due to concentrations used. Grossman^19^ did not observe effects at 100 µg/L but found significant anxiolytic effects at 250 µg/L. Our highest acute dose being 150 µg/L suggests that sedative effects of LSD may occur prior to anxiolytic effects which require higher doses. Although, Green and colleagues^20^ did find anxiolytic effects at 100 µg/L LSD with a group shoaling test. Our behavioural tests, as well as those conducted by Grossman^19^ were on individual zebrafish, suggesting that LSD may have a synergistic effect on zebrafish shoals.

Repeated microdosing had no effect on anxiety-like behaviour, boldness, or locomotion. There are a few potential explanations for these results. (1) Perhaps the individual tests we used were not sensitive enough to measure this change? Future studies could use group shoaling to investigate this and/or increase the dose of the LSD. (2) Zebrafish may have become tolerant to the repeated administration of LSD. Drugs cause many forms of tolerance (i.e., metabolic, pharmacodynamic, behavioural) that act via many mechanisms, including neuroadaptation. Pharmacokinetics of LSD have been investigated in rabbits, rats, and monkeys (*Macaca mulatta*), but not in zebrafish. The rate at which LSD is metabolized differs based on species. The half-life in a monkey is 100 min, in mice it is 7 min, and in humans, it is 175 min^21–24^ but the half-life in zebrafish is unknown. Also in zebrafish, repeated terpene exposure had no effect despite acute doses producing strong anxiolysis^25^.

The absence of a repeated LSD-mediated effect on behaviour is not a detriment to the potential of LSD as a therapeutic agent, and may be considered a benefit. Clinicians seek compounds with no behavioural side effects with repeated administration. The remaining question, which is arguably more important, is whether LSD may have a therapeutic effect? Future studies should use other behavioural tests such as the novel tank dive test which is a more sensitive test for detecting anxiety-like behaviour with some drugs^26^. In further research it will also be valuable to investigate whether microdoses of LSD can alleviate drug addiction in zebrafish, which are established models of withdrawal from ethanol^18,27^ and nicotine^28^. Microdoses of LSD may also be used as an agent to treat anxiety disorders and depression^29^ which may be more evident in zebrafish models. For example, there is a null mutation model of depression in zebrafish in which modification of the glucocorticoid receptor gene produces depression-like decreases in locomotion, and this can be alleviated with both anxiolytic/antidepressant selective serotonin reuptake inhibitors (SSRI), like fluoxetine, or a sedative anxiolytic drug like diazepam^30^. Treatment with repeated LSD exposure may have similar effects based on potentiation of serotonergic systems. Furthermore, environmental models of anxiety that involve the introduction of stressors^31,32^ may create a situation whereby LSD provides benefits. Recent rodent research has shown that repeated LSD administration is beneficial to stressed animals with no impact on those that are not stressed^33^. Taken together, the next step is to test LSD as a potential therapeutic agent with the above manipulations.

Overall, we have found no impact of repeated microdoses of LSD on anxiety-like behaviour, boldness, or locomotion under our experimental conditions. It is possible that with increased stress, withdrawal from drug addiction, or in a diseased state, repeated microdoses may be beneficial. The potential for LSD to act as a therapeutic agent thus shows promise, but future research is needed to determine its overall usefulness.

## Materials and methods

### Subjects

Adult zebrafish (~ 9 months old, n = 254) were bred in house in an established colony. 40 adult zebrafish (21 male and 19 female) were used in the acute dosing experiment, and 214 zebrafish (64 females and 150 males) were used in the repeated dosing experiment. All research was approved by the animal research ethics board (AREB) at MacEwan university (AUP: 101,853). All guidelines set by AREB and ARRIVE were followed. Fish were housed in a three-level housing system (Aquatic Habitats, FL, USA) in 3L or 10L tanks. The fish were bred in-house in the MacEwan Biology fish lab. Zebrafish in acute dosing were housed in four linked ZebTec habitats with racks and a centralized water treatment unit (Techniplast, Montreal, QC, Canada). This housing unit also used 3L and 10L housing habitats. All fish used for dosing and testing where stored in 3L tanks with breeding inserts with 12–15 fish per tank (~ 4.3 fish/L). Fish where dosed with the same fish in their housing tank. The fish were randomly selected into their housing tanks. The housing tanks were assigned their dosing schedule using a random number generator. pHset point was 7.1 and temperature had a set point of 28.5 °C. Zebrafish sex was determined using body shapes and colouration. For example, females are much rounder in the belly area with a silver belly^34,35^. The sex of the fish was determined after behavioural testing for both post-dose and withdrawal trials. The fish were kept on a 12-h day/night cycle^36^, with the lights turning on at 8:00 and the lights turning off at 20:00. The pH, temperature using an AquaController (Neptune systems, CA, USA), water conductivity using a DiST (HANNA, RI, USA), and the dissolved oxygen using a dissolved oxygen meter (Extach instruments, MA, USA) was monitored throughout the day. The temperature was maintained between 25–29 °C, and the pH was between 7.00–8.00, with the dissolved oxygen between 5.0–10.0. Fish were randomly distributed to LSD groups; 1.5 µg/L (n = 52), 15 µg/L (n = 54), 150 µg/L (n = 54), and control (n = 52). All tests were conducted on all the fish sequentially in a day, with 12–15 fish tested per day while maintaining the dosing time across all groups. All fish were naïve to experimental testing and pharmacology prior to this study.

### Drug administration

LSD (Sigma, ON, Canada) concentrations (1.5 µg/L, 15 µg/L, and 150 µg/L) were based on previous zebrafish research^19^. For the acute doses, LSD solutions (1.5 µg/L, 15 µg/L, and 150 µg/L and control) were prepared each day in 1L of tank water and kept at 25–29 °C^37^. For repeated dosing experiments, 3 L tanks were filled with 1 L of buffered reverse osmosis (RO) water. There were 4 replicates for each experimental group. The RO water was created in batches the night before dosing. The normal RO water was mixed in a container with 1.03 g of sodium bicarbonate (NaHCO_3_) to increase the pH to a range of 6.5–8^37^. When the buffered water was used for dosing, it was first warmed to 25–29 °C, and the pH was verified. The water was poured into the dosing containers from a height of 30 cm to increase dissolved oxygen (dO) to above 5 ppm, measured using a dissolved oxygen meter (ETECH instruments, MA, USA). The water conductivity of the dosing water was taken intermittently to ensure it was at an appropriate level (DIST by HANNA, RI, USA) ~ 220 to 260 µS/cm, which is within the acceptable range of 150 µS/cm and below 1700 µS/cm for zebrafish^37^. The dosing tanks were placed within a white grid-like enclosure (7A-C), thus preventing the fish from being startled from external visual stimuli. The fish were then moved from their housing tank using breeding inserts, thus minimizing potential anxiety due to repeated netting^18^. The dosage tanks were then set into a quadrant arena made from white corrugated plastic. Each section of the quadrant was a 10.25 cm × 10.25 cm space allowing ample room for the dosage tank. The quadrants were set on a seed starter heat mat (Ipower, CA, USA or Hydrofarm, CA, USA) to maintain a constant water temperature during dosing^37^. The fish were then dosed for 30 min using a stopwatch to monitor the time (3000 ADANAC by MARATHON, Ontario, Canada). Since other studies report no significant results with small doses of LSD (5–100 µg/L) for 20 min^19^, the time was increased to be consistent with other published work^38^. After the 30-min dosing period, the fish were returned to their regular habitat tanks and fed 1/8 tsp of flake food immediately after dosing (Gemma-micro-300, France). pH was retested and no change was noticed after the addition of LSD. All dosing was conducted in the first 8 h of the light cycle. At the start and end of every dosing day, the feeding activity, physical appearance, measurable signs (size, colour, eye abnormalities, posture in the water column, ventilation rate, gasping), movement, and body condition were observed to verify each fish was healthy during the dosing period.

### Behavioural testing

In acute experiments, fish were acclimated to the testing room for 30 min and were then dosed for 30-min before behavioural tests. In repeated dosing experiments, fish were tested immediately after the final day of dosing. After their final 30-min dose, the fish were moved to the testing room in their habitat tank and acclimated to the testing room for 30 min. All testing was completed during the light hours of the day/night cycle (8:00–20:00) typically from 9:00–18:00. Groups were tested in an alternating fashion to prevent any time inconsistencies between groups After the first set of testing, the fish were returned to their habitat and fed. After 7 days passed, the repeatedly exposed fish were tested again under otherwise identical procedures. The experimenter was not blinded for testing or dosing due to the complicated repeated dosing procedures and ongoing COVID-19 procedures at the time of experimentation.

### Open field test (OF)

**Figure 7:**
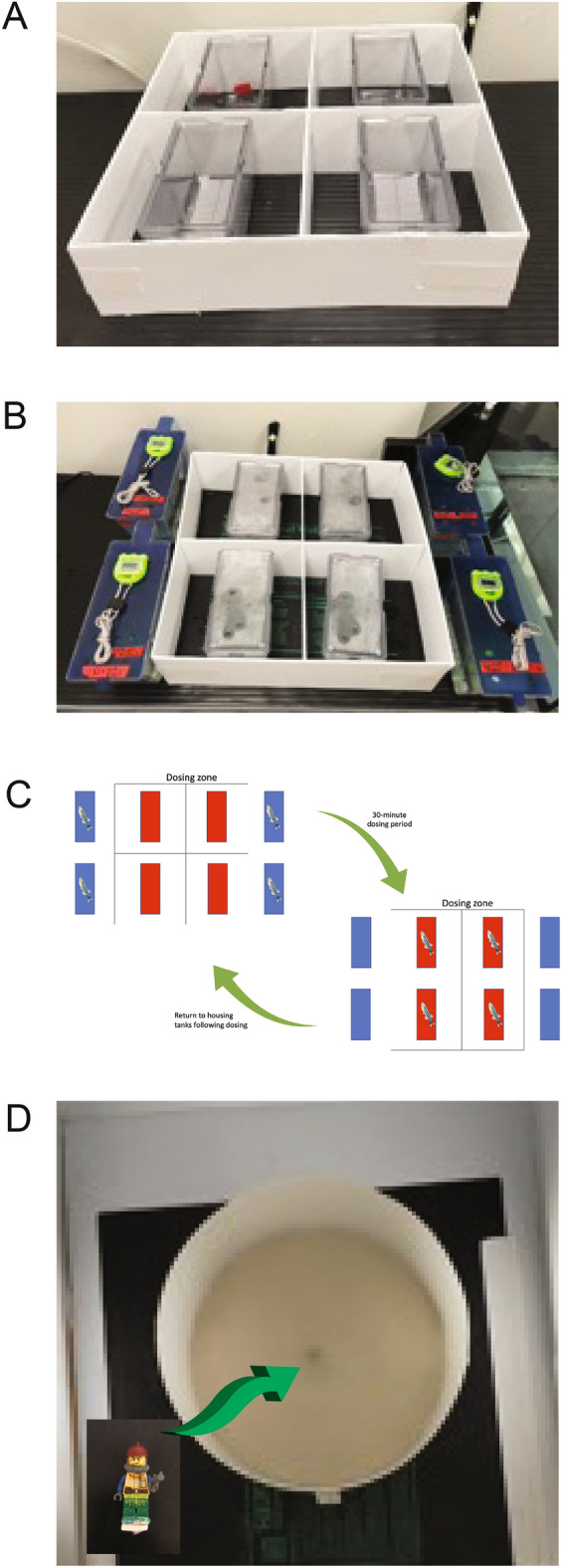
Dosing procedure and behavioural testing apparatus. (**A**) The dosing tanks placed in a white grid closure. (**B**) Dosing tanks during dosing. (**C**) Diagram of the dosing procedure. (**D**) Circular arena used in the OF and NOA tests. Inset is the Lego figurine added prior to the NOA.

Following the acclimation period, the fish were individually placed via the net, into the OF arena and allowed to swim from the net facing the center of the arena. Testing duration was 10 min. The circular arena had a diameter of 34 cm and a height of 16 cm (Fig. 7C). The arena was filled with 6 cm of habitat water and was placed on a heat mat to maintain the water temperature at 26–29 °C. Water was changed after every 4–6 fish, typically every 5^th^ fish, to ensure the dissolved oxygen was at an appropriate level and to maintain temperature^39^. During the testing period, the arena was at a light level of 28 cd/m^2^ (Calspot photometer, MI, USA). The OF test is validated to study anxiety-like behaviour and locomotion in zebrafish^40^.

### Novel object approach test (NOA)

After the 10-min OF test a novel object was introduced into the center of the arena. The object was a Lego figurine^39,41^, and was held in place using a small Velcro patch on its feet (Fig. 7D). The researcher placed the object in the center of the arena wearing a rubber glove and recorded behavior for another 10 min. This test is used to measure the tendency of a fish to explore the never-before-seen object, with an increase in time around the object related to higher boldness of the fish^27^. Approach to the novel object can be used to describe boldness many species including rainbow trout,^42^, the guppy (*Poecilia reticulata*)^43^, paradise fish^44^, and zebrafish^45^.

### Motion-tracking

The trials were captured using motion-tracking software, EthoVision XT (version 10; Noldus, Wageningen, Netherlands). The camera (Basler, PA, USA) used was located 1 m above the arena. The arena was divided into three zones using EthoVision. The three zones, thigmotaxis, transition, and center, were created virtually on the software program. The inner zone had a diameter of 4.5 cm and was located at the center of the arena. The transition zone had width of 2.75 cm from the inner zone to the thigmotaxis zone. Finally, the thigmotaxis zone was 4.5 cm wide, spanning from the transition zone to the outer wall of the arena. Dependent variables analyzed in both tests were the time spent in zones, velocity, high mobility, mobility, immobility, which was set to 5% threshold^46^, and meandering (the change of direction relative to the distance moved). The mobility was a calculation of the duration of the animal's movement through the arena. The set mobility threshold was above 60%, classified as high mobility, and below 5% as low mobility. Meandering was calculated by dividing the turn angle by the distance moved. After the test, the fish were placed in a 500 mL beaker filled with 300 mL of habitat water to allow for sexing of the fish^47^. The 300 mL was changed every 6 fish. After determining the sex of the fish, the fish were placed back into a 3 L habitat tank and fed their food post-test. One week after the post-dosage testing, the fish were once again tested. All data were interpolated in the track editor of EthoVision to fill in any missed samples during the trials.

### Statistical analysis

Normality was tested with the D'Agostino-Pearson test. Acute experiments were analyzed with an ordinary one-way ANOVA or Kruskal–Wallis test with a Tukey’s multiple comparison for parametric data and a Dunn’s multiple comparison for the nonparametric data. The nested ANOVA^48^ was used for the repeated experiment. A nested ANOVA is used in experiments in which the groups have two or more subgroups (replicates). If a data set did not pass the normality test it was then normalized. Each column was normalized individually using the smallest variable as 0 and the largest variable as 100. The significance level for the confidence intervals was 5% (*α* = 0.05) for all analyses. All data was analyzed with Prism and no outliers or data was removed (v. 9, GraphPad, San Diego, CA).

### Supplementary Information

Supplementary Information 1.

Supplementary Information 2.

Supplementary Information 3.

Supplementary Information 4.

## Data Availability

All data generated or analyzed during this study are included in this article’s supplementary files.
